# No Consequence for Lateral View X-Ray in Displaced Proximal Femoral Fractures in the Elderly

**DOI:** 10.3389/fsurg.2021.652528

**Published:** 2021-05-24

**Authors:** Christian Macke, Maic Werner, Lambert Herold, Olaf Krause, Tilmann Graulich, Jan-Dierk Clausen, Christian Krettek, Emmanouil Liodakis

**Affiliations:** ^1^Trauma Department, Hannover Medical School, Hannover, Germany; ^2^Institute for General Medicine, Hannover Medical School, Hannover, Germany

**Keywords:** proximal femoral fracture, elderly, lateral view, X-ray, operative treatment, DHS, proximal femoral nail

## Abstract

**Background:** Due to demographic changes, proximal femoral fractures (PFF) in the elderly rise constantly. The standard diagnostic tool is still the X-ray of the pelvis/hip in two planes. Our hypothesis was that the lateral-view X-ray has little influence on classification, planning of the operative procedure, and choice of implant in geriatric patients.

**Methods:** Retrospective analysis of all initial X-rays of PFF in geriatric patients (≥70 years) from May 2018 until August 2019 in a Level I Trauma center. Three experienced consultants categorized the fractures on the ap pelvis view and performed Garden and Pauwels classification as well as a two-staged classification displaced/nondisplaced [for femoral neck fractures (FNF)] or AO Classification [for intertrochanteric fractures (ITF)]. Afterward, they decided the operative strategy as well as implant choice [dynamic hip screw (DHS), intramedullary nail (IMN), or arthroplasty]. After 4 weeks, they categorized all fractures again with now available lateral view X-rays in a different order.

**Results:** Two hundred seven patients (146 female, 61 male; 70.5 vs. 29.5%) with 90 FNF and 117 ITF (43.5 vs. 56.5%) could be included. Age was 84.6 ± 6.9 years. The treatment was in 45 cases DHS, in 82 cases IMN, and for the other 80 cases arthroplasty. The interobserver reliability of the classifications were poor, except for the two-staged classification [Fleiss-κ ap view only = 0.708 (CI 95% 0.604, 0.812) vs. additional lateral = 0.756 (CI 95% 0.644, 0.869)]. Moreover, independent from the classification, there were no significant changes in management and choice of implant with additional lateral view.

**Conclusions:** Regarding our results, we consider the lateral view dispensable for standard X-ray of displaced PFF in geriatric patients. In nondisplaced fractures, it could be added secondary.

## Introduction

Proximal femoral fractures (PFF) in the elderly rise constantly and are a burden to our healthcare systems (1, 2). The standard diagnostic tool is still the X-ray of the pelvis/hip in two planes. Usually, the ap view is necessary for classification and evaluation of the fracture pattern, whereas the lateral view is performed to evaluate posterior comminution or posterior tilt in femoral neck fractures (FNF) (3).

Although there have been fast-track protocols providing adequate analgesia (4), especially the lateral view can be very painful for the patients. The contralateral leg has to be flexed at least at 90°, thus causing movement of the fracture site. Moreover, the lateral X-ray causes a relevant radiation dose (5).

Nonetheless, the lateral view is still considered to be necessary for classification, planning of the operative procedure, and choice of implant in all proximal femoral fractures.

Although there have been some studies with lateral-view X-ray in proximal femoral fractures, showing little advantage for the lateral view, none has focused explicitly on the geriatric population (6–8).

The aim of this study was to evaluate whether the lateral view is necessary for classification, planning of the operative procedure, and choice of implant in proximal femoral fracture patterns in the geriatric population.

## Materials and Methods

We retrospectively analyzed all initial X-rays of PFF in geriatric patients (≥70 years) from May 2018 until August 2019 in our Level I Trauma center.

All patients with operative treatment of these fracture types were included. The intraoperative diagnosis was set as the “gold standard.” Patients without X-ray of pelvis ap as well as lateral view were excluded. Another exclusion criterion was peri-prosthetic fractures.

All X-rays were taken in the emergency department prior to admission. The standard in our hospital is a pelvis ap view and a lateral view of the fractured site with the contralateral hip flexed >90°. The X-rays were extracted from our hospital PACS system.

General patient data was gathered from the patient records. Three experienced consultants (>10 years of expertise in PFF treatment) categorized the fractures on the ap pelvis view and performed Garden and Pauwels classification as well as a two-staged classification displaced/nondisplaced [for femoral neck fractures (FNF)] or AO Classification [for intertrochanteric fractures (ITF)] = group AP. Afterward, they decided the operative strategy in view of implant choice [dynamic hip screw (DHS), intramedullary nail (IMN), or arthroplasty]. After 4 weeks, they categorized all fractures again with now available ap and lateral view X-rays in a different order = group LAT.

Each author certifies that all investigations were conducted in conformity with ethical principles of research. Ethical approval was given through the institutional review board (IRB).

Statistical analysis was performed with SPSS 27.0 (SPSS Inc., Chicago, IL, USA). Categorical variables were expressed as the number and percentage. Fisher's exact test was used to evaluate differences in categorical values between groups. Inter- and intra-observer reliability was calculated using Fleiss–Kappa and Cohens–Kappa, respectively.

Statistical significance was considered with a two-tailed *p*-value of <0.05.

## Results

We could include 207 patients. The mean age was 84.6 ± 6.9 years (70–101 years). Most patients were female (*n* = 146/207, 70.5%), and these were more likely to have a femoral neck fracture than men (*p* = 0.094; Table 1).

**Table 1:** Distribution of fracture patterns of male and female patients.

**Parameter**	**Male (*n*, %)**	**Female (*n*, %)**	**All (*n*, %)**
Femoral neck fracture	21 (34.4)	69 (47.3)	90 (43.5)
Garden 1	3 (4.9)	8 (5.5)	11 (5.3)
Garden 2	3 (4.9)	8 (5.5)	11 (5.3)
Garden 3	7 (11.5)	20 (13.7)	27 (13.0)
Garden 4	8 (13.1)	33 (22.6)	41 (19.8)
Pauwels 1	4 (6.6)	7 (4.8)	11 (5.3)
Pauwels 2	3 (4.9)	11 (7.5)	14 (6.8)
Pauwels 3	14 (23.0)	51 (34.9)	65 (31.4)
Intertrochanteric fracture	40 (65.6)	77 (52.7)	117 (56.5)
31A1	18 (29.5)	34 (23.3)	52 (25.1)
31A2	12 (19.7)	32 (21.9)	44 (21.3)
31A3	10 (16.4)	11 (7.5)	21 (10.1)
Overall	61 (100.0)	146 (100.0)	207 (100.0)

With the lateral view available, the overall rate of correct classification improved (71.0 vs. 83.4%; *p* < 0.001). However, this was only due to a significant improvement in the minor displaced fracture patterns of Garden 1 and 2, as well as 31A1 and the nondisplaced FNF (see Table 2). None of the displaced fracture patterns (displaced FNF, Garden III and IV, 31 A2 and A3) improved significantly. There was no improvement regarding correct operative procedure (group AP: 84.5% vs. group LAT 80.5%; *p* = 0.073) or patient positioning (AP 76.3% vs. LAT 73.6%; *p* = 0.295) in any of the groups or fracture patterns.

**Table 2:** Percentage of correct diagnosis for proximal femoral fractures depending on AP view (AP) or ap and lateral view (Lat).

**Classification**	**AP (%)**	**Lat (%)**	***p*-value**
Garden 1	51.5	84.8	**0.007**
Garden 2	33.3	63.6	**0.026**
Garden 3	69.1	80.2	0.148
Garden 4	75.6	85.4	0.076
FNF nondisplaced	74.2	89.4	**0.041**
FNF displaced	97.5	97.1	>0.9999
A1	67.9	85.3	** <0.001**
A2	87.9	87.1	>0.9999
A3	66.7	81.0	0.104

*FNF, femoral neck fracture*.

*The bold values are statistically significant with p < 0.05*.

There were overall 14 DHS and 76 arthroplasties in the femoral neck fracture group. All of the Garden 3 and 4 fractures were treated with arthroplasty (Table 3).

**Table 3:** Operative procedure of femoral neck fractures depending on the Garden classification.

**Classification**	**DHS (*n*, %)**	**Arthroplasty (*n*, %)**
Garden 1	10 (90.9)	1 (9.1)
Garden 2	4 (36.4)	7 (63.6)
Garden 3	0 (0.0)	27 (100.0)
Garden 4	0 (0.0)	41 (100.0)
All	14 (15.6)	76 (84.4)

*DHS, Dynamic hip screw*.

For the Garden 1 fracture type, all patients except one were treated with DHS. The one patient with arthroplasty in this group was a patient with osteoarthritis of the hip (Kellgren–Lawrence Score 4) already planned for elective surgery.

In Garden 2 fractures, there were four DHS and seven arthroplasties. The three observers changed their classification in about 50% after lateral view X-ray, but that had only little effect on the operative procedure.

Inter-rater reliability was only moderate and without significant differences for Garden Classification [Fleiss-κ AP = 0.418 (CI 95% = 0.343–0.492) vs. LAT = 0.469 (CI 95% = 0.394–0.544)]. Inter-rater reliability for the operative procedure for femoral neck fractures was better [Fleiss-κ AP = 0.514 (CI 95% = 0.408–0.62) vs. LAT = 0.715 (CI 95% = 0.598–0.833)], but neither significant.

For a two-staged classification with nondisplaced vs. displaced femoral neck fractures, the correct overall rating improved from 91.9 to 95.2% with an additive axial view, but this was not significant (*p* = 0.161). Moreover, this improvement with the axial view available was due to the increase in correct rated nondisplaced fractures from 74.2 to 89.4%. The displaced fractures did not change at all with 97.5% correct rating without axial view and 97.1% with axial view.

The inter-rater reliability was substantial for this two-staged classification [Fleiss-κ AP = 0.708 (CI 95% = 0.604–0.812) vs. LAT = 0.756 (CI 95% = 0.644–0.869)].

Except one planned arthroplasty, all 31A3 fractures were treated with IMN (Table 4).

**Table 4:** Operative procedure for intertrochanteric fracture depending on the AO classification.

**Classification**	**DHS (*n*, %)**	**IMN (*n*, %)**	**Arthroplasty (*n*, %)**
31A1	29 (55.8)	21 (40.4)	2 (3.8)
31A2	2 (4.5)	41 (93.2)	1 (2.3)
31A3	0 (0.0)	20 (95.2)	1 (4.8)
All	31 (26.5)	82 (70.1)	4 (3.4)

*DHS, Dynamic hip screw; IMN, intramedullary nailing*.

Almost all of the 31A2 fractures were treated with an intramedullary nail, whereas the operative procedure varied in 31A1 fractures. Only the classification of the 31A1 fractures improved significantly (Table 2) with available lateral view, but an additional lateral view X-ray in any of these fracture patterns did not improve the ratings for the operative procedure.

The inter-rater reliability in intertrochanteric fractures for AO Classification was better with lateral view [Fleiss-κ AP = 0.396 (CI 95% = 0.325–0.468) vs. LAT = 0.564 (CI 95% = 0.49–0.639)], but only poor to moderate. Inter-rater reliability for the operative procedure in intertrochanteric fractures was slightly better [Fleiss-κ AP = 0.513 (CI 95% = 0.416–0.61) vs. LAT = 0.579 (CI 95% = 0.477–0.681)], but not significant.

Intra-rater reliability with Cohen's κ regarding all classifications was substantial (κ = 0.584–0.831) as well as for the operative procedure (κ = 0.68–0.805).

## Discussion

This study focused on the effect of the lateral-view X-ray in proximal femoral fractures in the elderly. As stated before, proximal femoral fractures in the elderly rise constantly and should be treated promptly (1). One necessary step to reach this goal is a correct diagnosis, which requires a classification of the fracture.

However, classification is a major problem. Masionis et al. (9) found only poor reliability for the common Garden and AO classification for femoral neck fractures but found good reliability if a simple two-stage classification of displaced vs. nondisplaced was used, and this was independent from the observer group (professors, trauma surgeons, or trauma residents). Another study of Crijns et al. (10) came to the same conclusion, showing only good to substantial agreement, if a two-staged classification was used. Our data supports this, as we could show the best inter-rater reliability in the two-staged classification in femoral neck fractures. Although all three observers are experienced consultants for traumatology and have a high intra-rater reliability, they were not able to perform more than a moderate inter-rater reliability in the Garden and AO classification, but this is according to the literature (11).

**Figure 1:**
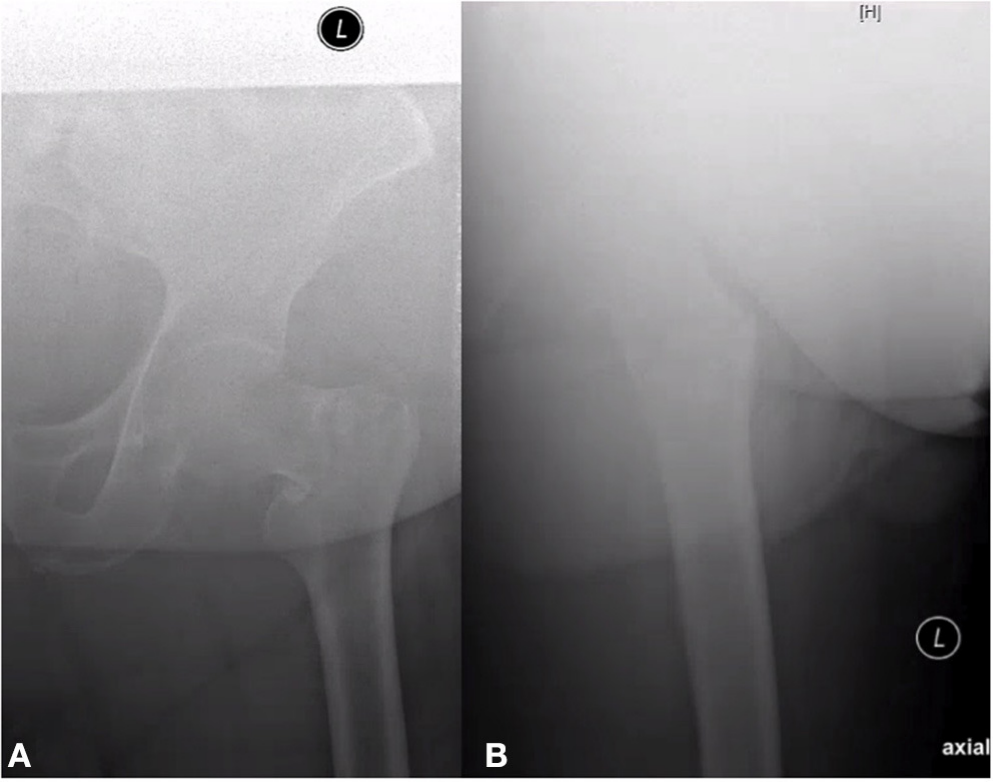
Ap **(A)** and axial **(B)** view of an intertrochanteric A1 fracture on the left side. Even with digital filter, the lateral view is not assessable due to obesity.

The lateral-view X-ray itself can be a problem. If the patient is obese, the quality of the X-ray decreases and evaluation and classification can be difficult (Figure 1). In fact, training of surgeons can improve their ability for performing and assessing intraoperative lateral X-rays in intertrochanteric fractures (12). However, though having better ratings for the fracture evaluation, the inter-rater reliability remains only fair in this study, which corresponds to our data for the intertrochanteric fractures.

One study suggests a prediction effect of the lateral view for displaced trochanteric fractures for intraoperative conversion from closed to open reduction (13). However, in their study only 11.3% were converted at a median waiting time to operation of 8 days, which limits the results relevantly and does not represent the everyday practice with these fractures. Furthermore, another study found good evidence for necessity for open reduction in displaced A2.3, A3.2, and A3.3 proximal femoral fractures anyway (14). In our study, an additional lateral view X-ray in any of this fracture patterns did not improve the ratings for conversion to open reduction or arthroplasty.

Overall, the correctness in evaluation of the fracture patterns was higher in the major displaced fractures and decreased in the minor displaced ones. This leads to the question if there are possibilities to improve especially the minor displaced fractures.

The Bristol hip view could be an option, as it has better results in recognizing occult fractures of the neck and possible dislocation grade than lateral view and there is no requirement for the patient to be moved (15).

Another possible tool for improving the evaluation of proximal femoral fractures in the ap pelvis view could be the traction-internal rotation X-ray (16, 17). Due to the retrospective study design of our study, we have not investigated it but thought of it as a good possibility as it improves especially the differentiation between displaced and nondisplaced fracture patterns.

There are limitations to this study. Each fracture is seen in our emergency department by an experienced trauma surgeon before admission to the ward, which limits the generalization to other hospitals or healthcare systems if only emergency registrars see the patient before operation. However, as mentioned above, experience seems to be of little influence for classification of these fracture types (11). Furthermore, as this is a retrospective study, the results should be verified by a prospective study.

## Conclusion

We could show in this study that the lateral-view X-ray in displaced proximal femoral fractures in the elderly is of little benefit. In fact, the correctness of classification increased in the minor displaced fracture types, but the operation procedure did not improve or change significantly. Therefore, we think the lateral view negligible for displaced fractures. In minor displaced fractures or in case of uncertainty, the lateral view could be added secondary or a CT scan should be performed.

## Data Availability Statement

The raw data supporting the conclusions of this article will be made available by the authors, without undue reservation.

## Ethics Statement

The studies involving human participants were reviewed and approved by Ethics Committee of Hannover Medical School, Hannover, number 8477_BO_K_2019. The patients/participants provided their written informed consent to participate in this study.

## Author Contributions

CM: study design and realization, data collection, analysis and interpretation of data, and writing of the manuscript. MW and LH: data collection, analysis and interpretation of data, and review of the manuscript. OK and TG: analysis and interpretation of data, review of the manuscript. J-DC: analyzing and interpreting data, reviewing manuscript. CK: review of the manuscript. EL: study design and realization, data collection, analysis and interpretation of data, review of the manuscript. All authors contributed to the article and approved the submitted version.

## Conflict of Interest

The authors declare that the research was conducted in the absence of any commercial or financial relationships that could be construed as a potential conflict of interest.

## Abbreviations

AP: group with only available pelvis ap view
DHS: dynamic hip screw
FNF: femoral neck fracture(s)
IMN: intramedullary nail
ITF: intertrochanteric fracture(s)
LAT: group with available pelvis ap view and additional lateral view
PFF: proximal femoral fracture(s).
